# Behaviour-based functional and dysfunctional strategies of medical students to cope with burnout

**DOI:** 10.1080/10872981.2019.1607506

**Published:** 2019-04-23

**Authors:** Joshua Welsh

**Affiliations:** Department of Medicine, Faculty of Medicine, Imperial College London, UK

**Dear Editor**,

I have read, with interest, the article by Erschens *et al* evaluating the strategies that medical students employ to cope with burnout [1]. Being a 5^th^ year medical student from Imperial College London I have personal experience of the approaches by medical students to help cope with burnout during examinations.

Medicine is an exciting and challenging career that requires individuals to be dedicated, hard working and able to cope with the many stressors that encompass the profession. This, coupled with the rigorous examination process throughout medical school, empowers students to develop strategies to prevent burnout. It is these behavioural strategies that can often determine an individual’s success during their time at medical school.

Cecil *et al* investigated the health behaviours that may predict burnout in medical students in the UK [2]. The health behaviours that appear to predict burnout in medical students include physical activity, year of study, gender and the institution of study. This illustrates that there are genetic and environmental factors that can predispose to burnout in students alongside behaviour-based strategies. Erschens [1], however, looked wholly at functional and dysfunctional behavioural approaches to deal with student burnout. Whilst the behaviours that were beneficial and those that were detrimental were identified, they appear to have overlooked the contribution of genetic and environmental factors in this process. A comparison with the impact of gender and institution of study on a student’s aptitude for coping with burnout would be noteworthy.

Nevertheless, a crucial aspect to any student’s development is being able to identify and tailor revision to their individual needs. The functional coping strategies that appear successful vary, from seeking social support from friends and family to engaging in regular physical activity. Those not partaking in such strategies may benefit from institutional support at their place of study. This was demonstrated by Greeson *et al* [3] who studied the impact of a mind-body medicine workshop for medical students and discovered that this strategy significantly boosted students ability to cope with the stress and emotional demands of medical school and encouraged social-support seeking behaviours.

To conclude, behaviour-based coping strategies to avoid burnout are fundamental to all medical students during their time at medical school. Managing the laborious revision periods is a complex and emotional task. Whilst some find the transition to medical school examinations seamless, others lack the coping mechanisms to balance their workload adequately and it is these individuals that are most at risk of burnout. Implementation of coping strategies such as mindfulness-based therapies, at an institutional level, would act as an excellent supplement to students seeking additional guidance or support on how best to cope with burnout at medical school.
